# Work of Breathing: Physiology, Measurement, and Diagnostic Value in Childhood Pneumonia

**DOI:** 10.3390/children11060642

**Published:** 2024-05-26

**Authors:** Israel Amirav, Aleeza Manucot, Jane Crawley, Sapir Levi

**Affiliations:** 1 Pulmonary Unit, Dana-Dwek Children’s Hospital, Tel Aviv 6423906, Israel; amirav@ualberta.ca; 2 Department of Medicine, University of Alberta, Edmonton, AB T6G 2R3, Canada; manucot@ualberta.ca; 3 Centre for Tropical Medicine & Global Health, University of Oxford, Oxford OX3 7LG, UK; jane.crawley@ndm.ox.ac.uk

**Keywords:** work of breathing, respiratory distress, pneumonia, clinical signs, review

## Abstract

In clinical practice, increased “work of breathing” (WOB) is used to rapidly identify the acutely ill child in need of immediate clinical care, and is commonly used to support a clinical diagnosis of pneumonia. However, this key clinical sign is poorly understood and inconsistently defined. This review discusses the physiology, measurement, and clinical assessment of WOB, highlighting its utility in the recognition of pneumonia in under-resourced settings, where access to diagnostic imaging may be limited.

## 1. Introduction

Pneumonia kills more children than any other infectious disease, claiming the lives of over 700,000 children under five every year, or around 2000 every day [1]. This includes around 190,000 newborns [1]. Almost all of these deaths are preventable [1]. Globally, there are over 1400 cases of pneumonia per 100,000 children, or 1 case per 71 children every year, with the greatest incidence occurring in South Asia (2500 cases per 100,000 children) and West and Central Africa (1620 cases per 100,000 children) [1]. The majority of these deaths occur in low- and middle-income countries (LMIC), where known risk factors such as poor sanitation and water quality, limited immunization, and undernutrition are prevalent [2]. Although early recognition and prompt, appropriate antibiotic treatment can reduce mortality from pneumonia [3] and minimize antibiotic overuse and the development of resistance, accurate diagnosis poses a significant clinical challenge. The diagnosis of pneumonia in resource-poor countries is usually made based on clinical signs. Though many of these signs (temperature, respiratory rate, oxygen saturation, and auscultatory findings) are easy to measure, they may not be specific enough to discriminate pneumonia from other illnesses [4]. Recent studies have found that a set of clinical signs collectively suggesting the presence of increased work of breathing (chest indrawing, intercostal/subcostal recession, grunting, head nodding, tracheal tug, nasal flaring, etc.) was associated with a higher likelihood ratio for radiologically confirmed pneumonia compared to other clinical signs [5,6]. This review article will examine the physiology, measurement, and clinical assessment of work of breathing (WOB), with particular emphasis on diagnosing pneumonia.

### 1.1. Physiology of WOB

WOB is defined as the energy needed to overcome the elastic and flow-resistive forces of the lung and inflate or deflate the lungs, chest wall, or both [7,8,9]. Examples of elastic forces are the lung elastic recoil and the surface tension of the alveoli, while flow-resistive forces are frictional forces between tissues and gas molecules [10]. Specifically, WOB is the “cumulative product of distending pressure and the given volume displaced during inhalation or exhalation” [9]. Stoller and Hill [11] introduced the concept of WOB as the energy required to overcome the lung’s elastic recoil, displace the chest wall and abdomen, and overcome airway resistance, lung viscosity, and inertia. Inertia refers to the resistance of any physical object to a change in velocity. As defined by most authors, total WOB is, therefore, the sum of elastic work, resistive work, and, though negligible, inertial work, which encompasses both inspiration and expiration [7,12,13].

Total WOB = Elastic WOB + Flow-Resistive WOB

Elastic WOB accounts for two-thirds of the total WOB, and flow-resistive WOB accounts for one-third [13]. Di Fiore and Carlo [12] define elastic work of breathing as the “work required to stretch the lungs and chest wall during a tidal inspiration” and resistive work of breathing as the “work required to overcome friction caused by lung tissue movement and gas flow through the airways”. Most of the elastic energy will be recovered during passive expiration, which is stored as elastic recoil, and the work performed during a single breath represents energy lost while overcoming resistive forces.

As disease or other external factors can change elastic and flow-resistive forces, total WOB will change accordingly, making it a potentially useful indicator of abnormal respiratory function [8].

### 1.2. Measurement of WOB

Since “work” is defined in physics as the product of pressure multiplied by volume, WOB can, therefore, be quickly and directly estimated by multiplying changes in pressure across the lung by changes in volume [7,14].

W = ΔPΔV,

wherein W = work of breathing, ΔP = change in pressure across lung, and ΔV = change in lung volume.

Transpulmonary pressure, the difference between alveolar and intrapleural pressure, can be multiplied by tidal volume to estimate total WOB [7,15]. This technique is convenient as an estimate because such respiratory parameters can be directly measured using pressure transducers, plethysmographs, and esophageal balloons.

**Figure 1:**
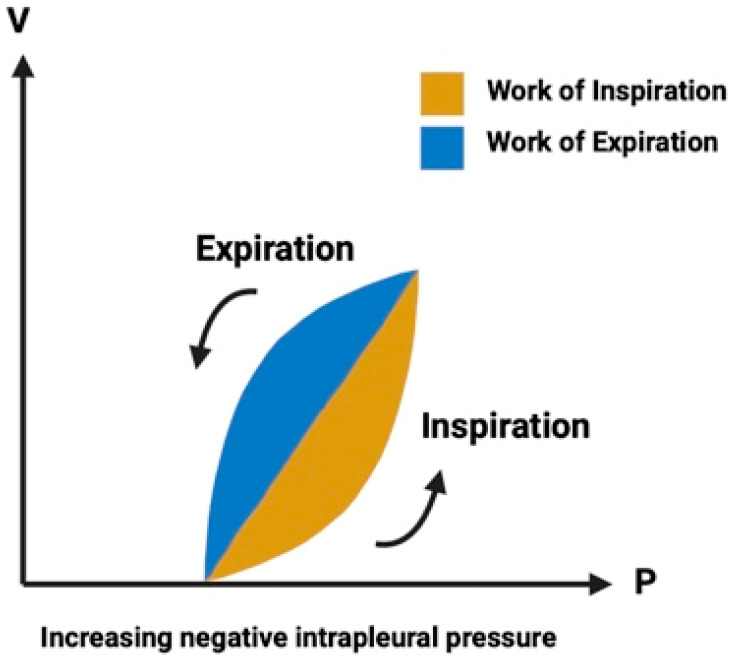
Normal work of the breathing cycle is shown on a diagram of the respiratory cycle. (V = Volume, P = Pressure).

A more accurate estimation of WOB uses the Campbell diagram, which depicts the relationship between pressure and volume during spontaneous breathing. Esophageal pressure, measured by balloon catheters, is commonly used to estimate intrapleural pressure (P). This value is plotted against tidal volume (V), and WOB is calculated by adding the areas between the resulting inspiration and expiration curves (Figure 1). Using the Campbell diagram can help understand pulmonary mechanics during obstructive airway diseases. These conditions are characterized by expiratory flow limitation, which may prevent the lungs from emptying to the relaxation volume during breathing, resulting in dynamic hyperinflation. When inhalation is initiated from a volume above relaxation volume, the inspiratory muscles must lower pleural pressure substantially. Dynamic hyperinflation thus represents an inspiratory threshold load that must be overcome to initiate inhalation and which will considerably broaden the inspiratory part of WOB as depicted in the diagram [16].

### 1.3. Current Technologies for Objective Measurement of WOB

Devices currently used to measure WOB in both inpatient and outpatient clinical settings incorporate technology based on the Campbell Diagram concept. Esophageal pressure is generally measured using a balloon catheter, and tidal volume using a flow sensor. WOB has been measured in adult patients with respiratory failure [17,18], chronic obstructive pulmonary disease (COPD) [18,19], and chronic heart failure [20], and in children with parenchymal lung disease [13] and bronchiolitis [21]. Estimating transpulmonary pressure and WOB using esophageal manometry is an effective tool for adjusting positive end-expiratory pressure (PEEP) in critically ill patients. Maintaining pressures within a specific range minimizes the risk of alveolar overdistention and lung injury.

In contrast to invasive esophageal balloon catheter techniques, respiratory inductance plethysmography (RIP) is a non-invasive respiratory monitoring device, first introduced by Cohn in 1977, that provides the measurement of volume expansion using recording bands wrapped around the thorax and abdomen [22]. By convention, the ribcage expands during inhalation, and there is a simultaneous outward movement of the abdomen. With a progressive increase in WOB, the ribcage lags behind the abdominal compartment, generating an asynchronous pattern of breathing known as thoracoabdominal asynchrony (TAA). TAA is present when there is increased respiratory resistance in the upper airway, lower airway, or lung tissue, low lung compliance, as seen in lung parenchymal disease (i.e., pneumonia), or increased chest wall compliance, as seen in infants and newborns, as well as patients with neuromuscular disease. Strang et al. [23] recently utilized the “pneuRIP” (Creative Micro Designs, Newark, DE, USA). This non-invasive, wireless device uses RIP to measure changes in volume between the ribcage and abdomen and derives various WOB indices. These WOB indices reported by the pneuRIP belts, including phase angle, respiratory rate (RR), and labored breathing index (LBI), are used to document the degree of WOB. The phase angle parameter quantifies the degree of TAA between the ribcage and abdominal compartments during breathing, and the LBI is a measure of the respiratory effort that is generated during TAA, reported as a ratio estimating WOB effort [23]. Specifically, Strang et al. found that the phase angle and LBI values differed markedly between healthy children and patients with neuromuscular disease [23]. Instantaneous measurements of the phase angle collected from the pneuRIP belts have also proved helpful in measuring response to treatment interventions in the rodent model [24], and real-time monitoring of WOB indices was used to adjust high-flow nasal cannula treatment in premature infants [25]. Recently, optoelectronic plethysmography (OEP) was utilized to detect increased WOB in infants with spinal muscular atrophy (SMA) and to assess the therapeutic respiratory MOH WOBI muscles’ effect of gene modifiers in these infants [26].

Both invasive and non-invasive techniques may have some limitations, including the potential discomfort or risks associated with invasive methods like esophageal balloon catheters and the possible accuracy issues with non-invasive methods like respiratory inductance plethysmography.

### 1.4. Increased WOB in the Diagnosis of Childhood Pneumonia

The World Health Organization (WHO) [27] clinical definitions of pneumonia and severe pneumonia were developed primarily for primary care and secondary level hospitals (e.g., district hospitals), guiding both inpatient and outpatient care. A child with cough or difficulty breathing plus fast breathing or lower chest wall indrawing is classified as a case of pneumonia [27], though other clinical signs (nasal flaring, wheeze, abnormal findings on chest auscultation) may also be present. A child fulfills the definition of severe pneumonia [27] if they have features of pneumonia plus ≥1 of central cyanosis or oxygen saturation <90%; severe ‘respiratory distress’ or increased WOB (IWOB); or a general danger sign, namely, unable to drink, lethargy or coma, or convulsions. It is clear, therefore, that this non-specific clinical definition of severe pneumonia also includes severe disease from various other infectious and non-infectious causes. Table 1 provides definitions for all of these clinical signs and compares the sensitivity and specificity of each for radiologically confirmed pneumonia.

IWOB is defined as grunting, chest indrawing, or head nodding in infants, due to the use of accessory muscles of respiration [38,39,40]. In practice, however, healthcare workers generally rely on a subjective assessment of IWOB, based on a constellation of these clinical signs. The term ‘increased WOB’ is often used interchangeably with ‘respiratory distress’, yet the underlying pathophysiology of the two conditions may differ. Respiratory distress usually occurs when there is impaired air exchange that leads to decreased ventilation and oxygenation, and IWOB is the observed compensation. However, these terms are by no means interchangeable. For example, in severe malaria and other conditions, respiratory distress or ‘deep breathing’ is associated with metabolic acidosis and poor prognosis, whereas IWOB is not associated with metabolic acidosis [41,42].

A systematic review by Shah et al. [5] concluded that clinical features of IWOB (grunting, flaring, and retractions) outweighed the importance of tachypnea and auscultatory findings in the diagnosis of pneumonia, primarily because they had the highest specificity for radiologically confirmed pneumonia. Signs of IWOB also had the highest positive likelihood ratio (LR, 2.1 [95% CI, 1.6–2.7]) for pneumonia compared to other physical examination findings (temperature–LR, 1.7; tachypnea–LR, 1.5; auscultation–LR, 0.88–1.4) [6]. There were a few limitations to that systematic review. The study included a limited number of diagnostic studies (23 prospective cohort studies), which may not fully represent the diversity of clinical presentations and diagnostic challenges in pediatric pneumonia. In addition, other factors such as comorbidities, environmental factors, or healthcare settings may have also affected the results. Nevertheless, in LMIC settings, this systematic review concluded that IWOB and hypoxemia are highly indicative of pneumonia and are therefore of considerable clinical value in areas where chest radiography is unavailable [5]. This further emphasizes the importance of assessing for signs of IWOB to improve early detection of pneumonia, reduce mortality, and prevent antibiotic overuse.

Recognizing clinical signs of increased WOB is also important in neonates diagnosed with neonatal respiratory distress syndrome. Parkash et al. [43] studied 205 neonates, ages 0 to 28 days, with respiratory distress, caused mainly by pneumonia. They found that 100% of the neonates had a respiratory rate >60 breaths per minute, nasal flaring, and subcostal retractions, while 60.9% had grunting. Similarly, Sabzehei et al. [44] found intercostal retraction (75.3%), tachypnea (67.7%), and grunting (61.3%) to be the most common symptoms of neonatal respiratory distress. Mlay and Manji [45] found that *grunting* was the clinical sign most significantly associated with increased mortality among infants with severe respiratory distress. These studies further highlight the importance of recognizing signs of increased WOB, as they are strongly suggestive of severe illness, respiratory distress, and increased mortality.

## 2. Discussion

The rationale for assessing the work of breathing in childhood pneumonia is to identify patients who are at high risk of respiratory failure and require urgent intervention, including oxygen therapy and referral to a higher-level facility. Assessing WOB may contribute not only to triage and risk assessment but also to diagnosis and assessing response to treatment, particularly in resource-limited areas. This review has suggested that increased work of breathing is a sensitive parameter that can help clinicians predict pneumonia. Furthermore, there is evidence for the consequences when WOB is not appropriately assessed (e.g., missed diagnoses, delayed referral, poorly directed treatment, excess mortality).

Whether the work of breathing itself is the most important critical parameter to evaluate or whether investigating the cause of increased work of breathing is necessary, both approaches appear important and complementary. Assessing the work of breathing can provide valuable information about the severity of respiratory difficulties, but investigating the underlying cause, such as bacterial or viral pneumonia, can guide appropriate treatment and help prevent further deterioration.

Currently, healthcare providers in LMICs use standardized case management guidelines called the Integrated Management of Childhood Illnesses (IMCI) guidelines developed by the World Health Organization (WHO) for childhood pneumonia care [27]. While focusing on subjective clinical signs, the IMCI guidelines for pneumonia diagnostic criterion performs with low specificity, resulting in antibiotic overtreatment. We believe that by following appropriate RCTs, objective measurements of WOB could be easily integrated into the guidelines and enhance their performance.

In resource-limited areas, healthcare professionals can develop proficiency in detecting increased work of breathing to aid in the clinical diagnosis and management of childhood pneumonia. The use of work of breathing as a sensitive parameter can be particularly valuable in these settings where alternative methods of assessment may be limited. With appropriate training and support, healthcare workers in these settings can be empowered to accurately detect early signs of respiratory distress. This can prompt appropriate treatment initiation and referral to higher-level facilities as needed. To further assist decision-making in pneumonia, we are recently developing an innovative, objective tool to identify IWOB through depth imaging and artificial intelligence (AI) technologies (MOH_2019-03-14_006019). Machine learning (ML), an emerging technology within the field of AI, is used in this work to create an algorithm to improve the detection of IWOB by overcoming the intrinsic limitations of human perception and bias, reducing errors, and improving diagnosis and risk predictions. Our ML-based models are first trained using large multi-national repositories of data from children presenting with clinically annotated IWOB and healthy children. Once trained, the ML model can then use data from other individuals to determine their probability for that event. Objective identification of increased work of breathing will serve to assist in the diagnosis and prediction of severe childhood pneumonia.

Integrating AI-based technologies with mobile phones to identify IWOB has the potential to be more effective than human senses in detecting smaller or more subtle clinical differences. There are more mobile phones than people in the world [46], and two out of three mobile users live in the developing world. Given the acceptability, widespread use, and effectiveness of mobile technology interventions, integrating objective WOB measurements in mobile phones can expand access to medical services in resource-constrained, rural, or remote settings where highly trained and experienced healthcare providers may not be available. Additionally, these technologies can lead to substantial cost savings [47].

The benefits of artificial intelligence in pneumonia include improving human performance, democratizing medical knowledge and excellence, automating repetitive tasks, and optimizing the allocation of limited resources [48].

A few limitations of this review must be acknowledged. We did not address the potential variations in diagnostic criteria or practices across different regions or healthcare systems. In addition, the review did not explore the cost-effectiveness or feasibility of implementing the identified parameters of WOB in real-world clinical settings. These economic and practical constraints, the cost of equipment, maintenance, and the need for trained personnel may all influence the generalizability of the findings.

## 3. Conclusions

We have reviewed the physiology, measurement, and clinical assessment of WOB to understand its role in diagnosing pneumonia in children. Improved understanding of the components of increased WOB may lead to the development of novel and AI-based clinical tools that could facilitate standardized recognition, thereby helping healthcare providers identify and treat children with pneumonia in a timely manner.

## Figures and Tables

**Table 1:** Clinical features of pneumonia and severe pneumonia in children.

Clinical Sign	Definition	Sensitivity/Specificity for Radiologically Confirmed Pneumonia
Grunting	Abnormal, repetitive, short upper respiratory tract sounds that can be heard during expiration [28,29].	Of all the features of increased WOB, *grunting* was most specific for pneumonia [30].Grunting was identified as one of the clinical signs significant (*p*-value 0.038) for radiologically confirmed pneumonia in children (1–16 years) presenting with suspicion of pneumonia [31].
Nasal Flaring	The opening and widening of the nostrils while breathing [32]	Subjects with and without pneumonia differed significantly on *nasal flaring*, with a *p*-value of 0.001 [33]. A combination of *nasal flaring*, tachypnea and hypoxia, had the highest specificity of 0.98 for pneumonia [33].
Chest Retractions/Indrawing	Inward movement of the chest or lower chest wall as a child breathes in [5,27] is often associated with supraclavicular, intercostal, and/or subcostal retractions [29].	Shamo’on et al. [34] found that *chest indrawing* was frequently observed with high specificity and sensitivity in pneumonia. Retractions were identified as one of the clinical signs significant (*p*-value 0.047) for radiologically confirmed pneumonia in children (1–16 years) presenting with suspicion of pneumonia [33].
Tachypnea	>60 breaths/min at 0 to 2 months of age, >50 breaths/min at 2 to 12 months, >40 breaths/min for 1 to 4 years of age, and >30 breaths/min for greater than 5 years of age (WHO criteria)	Durbin and Stille [30] and Shamo’on, Hawamdah, Haddadin, and Jmejan [34] identified *tachypnea* as the most sensitive and specific sign of pneumonia. In children < 5, tachypnea had a sensitivity of 74% and a specificity of 67% for radiologically confirmed pneumonia [35].
Head Nodding	Downward nodding toward the chest each time a child breathes, as a result of accessory muscle usage during breathing [36].	Observed head nodding presented a low sensitivity (0.11) and a high specificity (0.94) for children (0–59 months) with radiologically confirmed pneumonia across 41 shared datasets [37].In children with confirmed pneumonia (2–59 months), the presence of head nodding demonstrated a sensitivity of 59% and a specificity of 94% to predict hypoxemia [38].

## Data Availability

Not applicable—no data were used in this review.

## Abbreviations

WOB	Work of breathing
ED	Emergency department
